# Case Report: Durable complete response to antigen-specific cytotoxic T lymphocyte therapy in advanced EGFR-TKI resistant lung adenocarcinoma: a case of adoptive cellular immunotherapy overcoming acquired targeted resistance

**DOI:** 10.3389/fimmu.2025.1637165

**Published:** 2025-09-01

**Authors:** Chenghao Fu, Chengyu Bian, Weiyou Zhu, Haonan Du, Linrui Han, Jian Zhu, Jun Wang, Lei Cao

**Affiliations:** ^1^ Department of Thoracic Surgery, The First Affiliated Hospital of Nanjing Medical University, Nanjing, Jiangsu, China; ^2^ Department of Oncology, The First Affiliated Hospital of Nanjing Medical University, Nanjing, Jiangsu, China; ^3^ Department of Thoracic Surgery, Shanghai Pulmonary Hospital, School of Medicine, Tongji University, Shanghai, China; ^4^ Department of Thoracic Surgery, The First Affiliated Hospital of Xi’an Jiaotong University, Xi’an, Shanxi, China; ^5^ Department of Thoracic Cardiovascular Surgery, General Hospital of Central Theater Command of the People’s Liberation Army, Wuhan, Hubei, China; ^6^ Department of Oncology, The Affiliated Suqian First People's Hospital of Nanjing Medical University, Suqian, Jiangsu, China

**Keywords:** ACTL, EGFR-TKI, resistance, advanced NSCLC, cellular immunotherapy

## Abstract

Adoptive cell therapy (ACT), an important component of tumor immunotherapy, achieves precise anti-cancer effects by reinfusing *in vitro*-processed immune cells, providing a new option for advanced tumor patients with resistance to chemotherapy or targeted therapy. Among them, antigen-specific cytotoxic T lymphocyte (ACTL) therapy innovatively integrates the natural expansion advantage of tumor-infiltrating lymphocytes (TILs) and the precise antigen-presenting mechanism of recombinant adeno-associated virus-transfected dendritic cells (rAAV-DCs), becoming a research focus. This case report describes a patient with IVa stage advanced lung adenocarcinoma with multiple intrapulmonary metastases carrying an epidermal growth factor receptor (EGFR) exon 19 deletion mutation. After receiving treatment with the third-generation EGFR-tyrosine kinase inhibitor (EGFR-TKI) drug osimertinib, the patient developed acquired resistance with unknown mechanisms and was in an immune-suppressed state. Subsequently, the patient received ACTL therapy and ultimately achieved a clinical complete response (cCR) and maintained it for six years of follow-up. This case is the first to report that ACTL therapy has achieved “clinical cure” in a patient with acquired EGFR-TKI resistance, indirectly suggesting the underlying mechanism of this therapy in reshaping the tumor immune microenvironment (TME). It illustrates that ACTL, as a novel cellular immunotherapy with both target precision and immune balance, has demonstrated potential in overcoming targeted resistance in advanced lung cancer and inducing deep remission.

## Introduction

Lung cancer, as the malignant tumor with the highest global incidence and mortality, has always been a research focus in the field of oncology (1). Epidermal growth factor receptor (EGFR) mutation is the most common driver gene mutation in non-small cell lung cancer (NSCLC) (2). The third-generation EGFR-tyrosine kinase inhibitor (EGFR-TKI) has become the standard therapy for advanced EGFR-mutated NSCLC due to its dual inhibitory effect on sensitive EGFR mutations and T790M resistance mutations (3). However, due to complex mechanisms such as tumor heterogeneity, compensatory activation of signaling pathways, and immune escape, almost all patients treated with EGFR-TKIs will eventually develop acquired resistance, leading to disease progression (4). The mechanisms of acquired EGFR-TKI resistance are complex, still remaining unknown in approximately 50% of patients, and their molecular basis is unclear (5). For such patients, second-line treatment options are limited, and more effective clinical intervention is urgently needed.

In recent years, immunotherapy has become an indispensable core component of comprehensive lung cancer treatment. Among the many modalities, adoptive cell therapy (ACT) has emerged as a highly promising development direction due to its characteristic of precise tumor cell killing. ACT breaks through tumor immune escape barriers by expanding or modifying patients’ autologous immune cells *in vitro*, making it particularly suitable for refractory cases with highly heterogeneous tumor microenvironments (TME) after failed targeted therapy (6). As a cutting-edge technology in the field of ACT, antigen-specific cytotoxic T lymphocyte (ACTL) therapy uses recombinant adeno-associated virus (rAAVs) to infect dendritic cells (DCs), efficiently present tumor-associated antigens, and then activates and expands cytotoxic T lymphocytes (CTLs) *in vitro* for reinfusion into the body (7).

This case report describes a patient with stage IVa lung adenocarcinoma carrying an EGFR exon 19 deletion mutation. After developing acquired resistance with unknown mechanisms during first-line treatment with osimertinib, the patient received ACTL therapy and ultimately achieved a clinical complete response (cCR) and maintained it for 6 years of follow-up. This case aims to provide important clinical evidence and innovative ideas for the treatment of advanced lung cancer, particularly in patients with targeted therapy resistance.

## Case presentation

**Figure 1:**
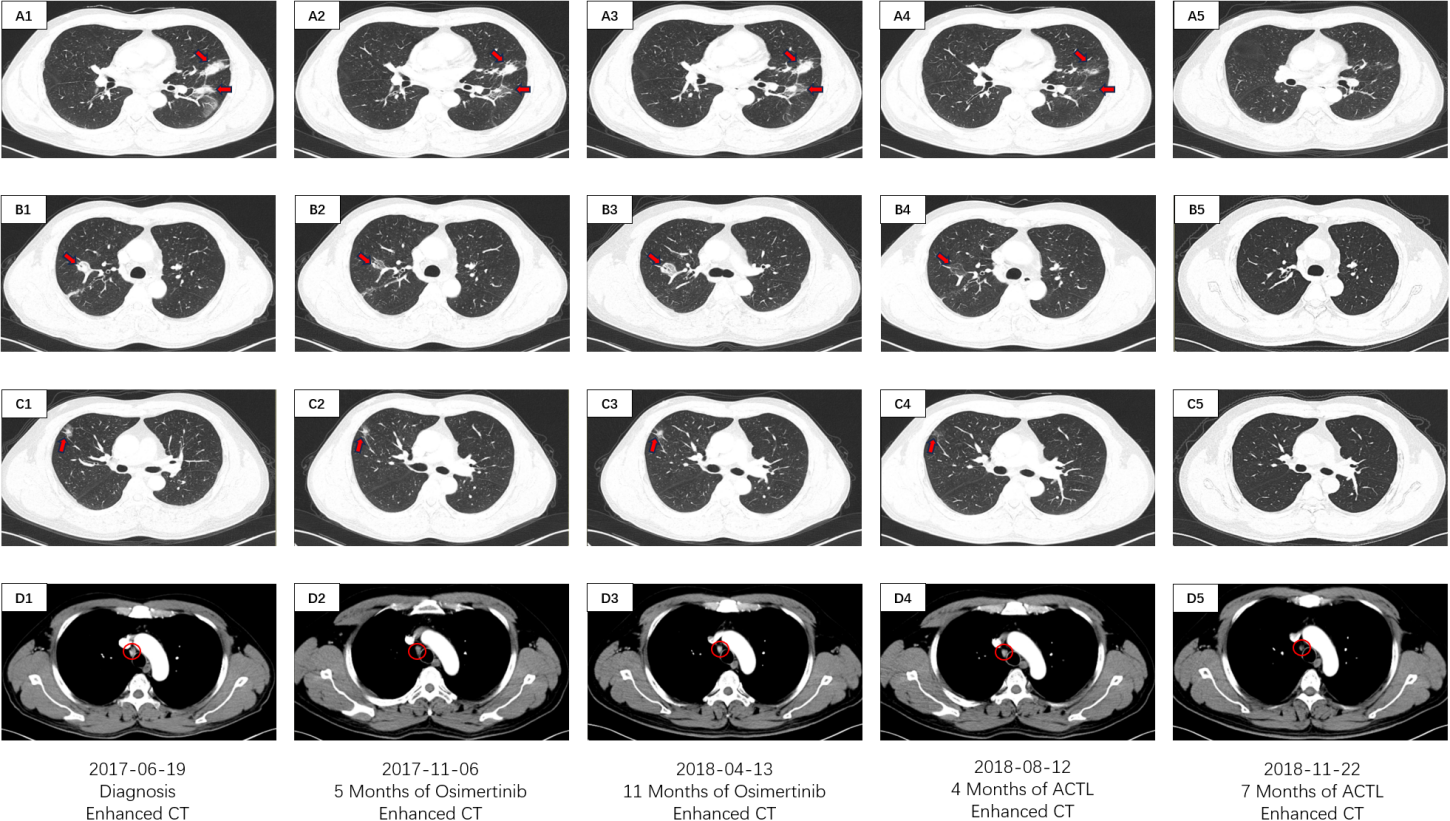
CT imaging assessment of lung lesions and mediastinal lymph node throughout the course of disease corresponding to their respective time points. **(A1–D1)** CT imaging of the lung window **(A1–C1)** and the mediastinal window **(D1)** on June 19, 2017; **(A2–D2)** CT imaging of the lung window **(A2, B2, C2)** and the mediastinal window **(D2)** on November 06, 2017; **(A3–D3)** CT imaging of the lung window **(A3–C3)** and the mediastinal window **(D3)** on April 13, 2018; **(A4–D4)** CT imaging of the lung window **(A4–C4)** and the mediastinal window **(D4)** on August 12, 2018; **(A5–D5)** CT imaging of the lung window **(A5–C5)** and the mediastinal window **(D5)** on November 22, 2018. **(A1–5)** images manifests the lesions of the left lobe in the CT lung window. **(B1–5)** images manifests the inner lesion of the right middle lobe in the CT lung window. **(C1–5)** images manifests the outer lesion of the right middle lobe in the CT lung window. **(D1–5)** images manifests the mediastinal lymph nodes in the CT mediastinal window.

**Figure 2:**
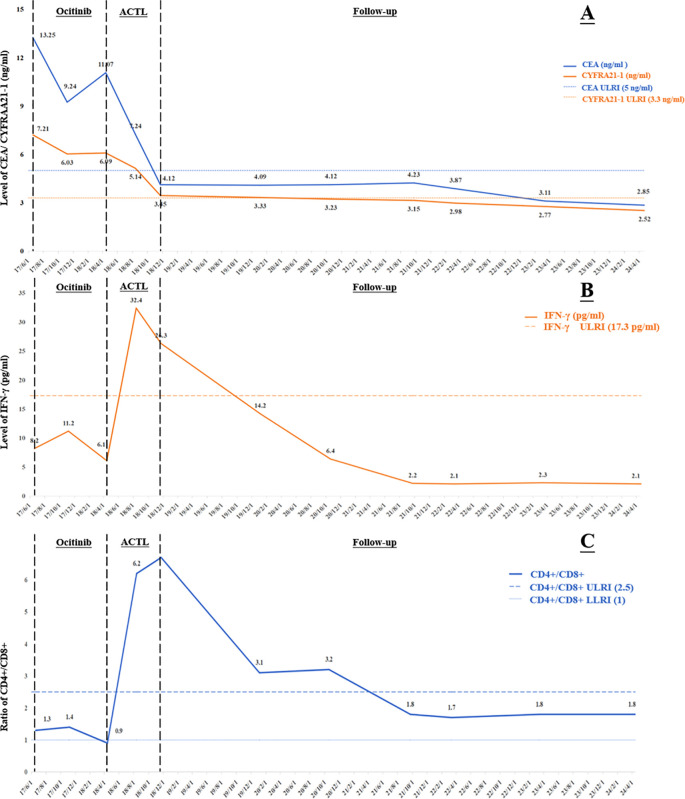
Change curves during treatment of: **(A)** tumor markers; **(B)** the cytokine index IFN-γ; **(C)** the lymphoid subset detection index CD4^+^/CD8^+^. CEA, carcinoembryonic antigen; CYFRA 21-1, cytokeratin 19 fragment; IFN-γ, Interferon-γ; ULRI, Upper limit of reference interval; LLRI, lower limit of reference interval; ACTL, antigen-specific cytotoxic T lymphocyte.

On June 19, 2017, a 53-year-old male patient presented with a 1-month history of persistent dry cough, intermittent expectoration of yellowish-white mucoid sputum, and nonspecific chest discomfort. The patient had no family history of malignant tumors, no smoking history, and no clear exposure history to environmental carcinogens. Laboratory tests indicated a significant inflammatory response: white blood cell count was 15.8×10^9^/L (predominantly neutrophils), and C-reactive protein was 177.75 mg/L. Contrast-enhanced chest computed tomography (CT) revealed multiple intrapulmonary space-occupying lesions: a main lesion crossing the interlobar fissure in the left lung, a 39.4 mm×21.5 mm nodule in the upper lung closely adjacent to the interlobar fissure, with obvious traction on the lateral pleura, and a 41.5 mm×23.8 mm purely solid nodule in the lower lung with ground-glass cord shadows between it and the lateral pleura. A 25.3 mm×22.6 mm purely solid nodule in the inner zone of the right middle lung showed thickened and traversing blood vessels within the lesion, with obvious solid traction to the lateral pleura. A 20.4 mm×14.5 mm mixed ground-glass nodule in the outer zone of the right middle lung had surrounding spiculation signs. Additionally, an abnormally enlarged lymph node (16.3 mm×9.36 mm) was observed in the mediastinum. Bilateral multiple nodular opacities were present, with multiple local cord-like and grid-like changes, highly suggestive of diffuse pulmonary metastasis with mediastinal lymph node metastasis on imaging (**Figures 1A1–D1**). Systemic evaluation including contrast-enhanced abdominal CT, bone scan, and contrast-enhanced cranial magnetic resonance imaging (MRI) revealed no distant metastases. Tumor markers showed abnormally elevated CEA level at 13.25 ng/ml (reference range: 0–5 ng/ml) and CK-19 fragment (CYFRA21-1) level at 7.21 ng/ml (reference range: 0–3.3 ng/ml) (**Figure 2A**). Cytokine/lymphocyte subset testing showed that IFN-γ levels and CD4^+^/CD8^+^ ratio were within normal ranges (**Figures 2B, C**).

To clarify the pathological nature, the patient underwent CT-guided lung lesion biopsy on June 23, 2017. The pathological results indicated adenocarcinoma. Immunohistochemical results showed positive staining for cytokeratin 7 (CK7), thyroid transcription factor-1 (TTF-1), and napsin A; negative staining for cytokeratin 20 (CK20), P40, and P63. Next-generation sequencing (NGS) revealed an EGFR exon 19 deletion mutation (EGFR 19 del), with no other driver gene mutations detected. The histological, immunohistochemical, and driver gene mutation status analyses of the sampled lesions in the left and right lungs were highly consistent. After multidisciplinary consultation by the departments of medical oncology, pathology, radiology, and thoracic surgery in our hospital, the patient was diagnosed with stage IVa lung adenocarcinoma with bilateral multiple intrapulmonary metastases and mediastinal lymph node metastasis (cT4N3M1a, stage IVa).

The patient had poor baseline physical tolerance, and targeted therapy was chosen. Considering their driver gene mutation status, the third-generation EGFR-tyrosine kinase inhibitor (EGFR-TKI) osimertinib was ultimately determined as the first-line treatment regimen. In the early stage of treatment, the patient’s condition showed remission, and they self-reported improvement in cough and sputum symptoms. A contrast-enhanced chest CT on November 6, 2017, showed that each intrapulmonary lesion had shrunk to a certain extent, with solid components showing a “ground-glass-like” dissipating trend; the solid traction between the lesions and the pleura was also relieved to varying degrees. The lymph node shrank to 11.6 mm×8.1 mm (**Figures 1A2–D2**). The CEA level decreased to 9.24 ng/ml, and the CYFRA21–1 level decreased to 6.03 ng/ml (**Figure 2A**). This indicated that osimertinib treatment was effective.

However, the patient subsequently gradually complained again of aggravated cough and chest discomfort. A contrast-enhanced chest CT on April 13, 2018, showed that the lesions in the upper and lower lobes of the left lung had solid components expanding to varying degrees; the lesion in the inner zone of the right middle lobe showed significant expansion in scope and solidity. The size of the lymph node was similar to before, but the center was slightly more enhanced than before (**Figure 1A3–D3**). The CEA level rebounded again to 11.07 ng/ml, and the CYFRA21–1 level rose to 6.09 ng/ml (**Figure 2A**). Imaging progression, tumor marker rebound, and symptom deterioration collectively indicated the occurrence of acquired resistance to osimertinib. We performed tissue biopsy combined with NGS, and multiple EGFR-TKI resistance-related mutations including T790M, Mesenchymal-epithelial transition factor (MET) amplification, Kirsten rat sarcoma viral oncogene homolog (KRAS) mutation, small cell transformation-related genes, etc., were all negative, indicating that the patient had acquired EGFR-TKI resistance with unknown mechanisms. At the same time, the patient’s immune indices showed significant changes, with IFN-γ decreasing from the previous 11.2 pg/ml to 6.1 pg/ml (reference range: 0–17.2 pg/ml), and the CD4+/CD8+ ratio decreasing from 1.4 to 0.9 (reference range: 1–2.5) (**Figures 2B, C**). This indicated a state of low immune function and overall immune suppression at this time.

In response to disease progression, the team considered adjusting the treatment regimen. After full communication between the medical team and the patient, the patient chose to receive ACTL adoptive immune cell therapy at our center. Based on the patient’s blood tumor marker test results, ACTL-targeted cellular immunotherapy with dual-target antigens of CEA and CK-19 (complete protein precursor of CYFRA21-1) was initiated. ACTLs were generated from peripheral blood monocytes via rAAV−transfected DCs presenting CEA and CK-19, followed by ex vivo activation and expansion in cytokine−supplemented media [interleukin-2 (IL−2) and granulocyte-macrophage colony-stimulating factor (GM−CSF)]. Rigorous quality control includes viability, sterility, and immunophenotype. Given the resistance to osimertinib, targeted drugs were suspended, and ACTL treatment was administered alone, with infusions every two weeks.

After ACTL treatment, the patient’s subjective symptoms improved. A contrast-enhanced chest CT just four months later showed surprising lesion resolution in multiple sites of both lungs. The lesions in the upper and lower lobes of the left lung shrank by 87% and 82%, respectively, with almost complete disappearance of solid components, leaving only partial ground-glass opacities, and the traction to the pleura disappeared. The solid components of the two lesions in the middle lobe of the right lung completely vanished, leaving only faint ground-glass-like contour shadows. The lymph node regressed to 9.21 mm×5.21 mm with reduced density (**Figures 1A4–D4**). Tumor marker levels decreased, with the CEA level dropping to 7.24 ng/ml and the CYFRA21–1 level decreasing to 5.14 ng/ml (**Figure 2A**). According to the Response Evaluation Criteria in Solid Tumors version 1.1 (RECIST 1.1) (8), this was determined as partial response (PR). Notably, the patient’s immune indices significantly improved simultaneously, with the IFN-γ level surging to 32.4 pg/ml and the CD4^+^/CD8^+^ ratio rising to 6.2, indicating that the body’s immune status had shifted from suppression to activation and from a previous low-functioning state to a high-functioning state (**Figures 2B, C**).

Treatment continued until November 22, 2018, when the patient’s symptoms completely disappeared. Contrast-enhanced chest CT showed that all lesions in both lungs had vanished; the lymph node shrank to a normal size of 4.02 mm×2.21 mm (**Figures 1A5–D5**). The CEA level was 4.12 ng/ml, and the CYFRA21–1 level was 3.45 ng/ml (**Figure 2A**). According to the RECIST 1.1 criteria, this was judged as clinical complete response (cCR). The follow-up has lasted more than 6 years to date. Imaging remains normal with no evidence of recurrence or progression, and tumor markers have remained normal, with CEA at 2.85 ng/ml and CYFRA21–1 at 2.52 ng/ml on April 22, 2024 (**Figure 2A**). The patient has maintained a stable and long-term cCR status. This case demonstrates the remarkable efficacy of ACTL cellular immunotherapy in advanced lung cancer patients with EGFR-TKI acquired resistance, providing new treatment insights for similar refractory cases.

**Figure 3:**
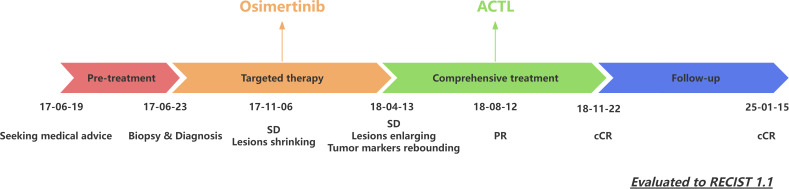
Patient’s treatment timeline. ACTL, antigen-specific cytotoxic T lymphocyte; SD, stable disease; PR, partial response; CR, complete response; RECIST, Response Evaluation Criteria in Solid Tumors.

The patient’s treatment timeline is shown in **Figure 3**. All infusion procedures were performed strictly in accordance with institutional standard operating procedures. Vital signs were continuously monitored during each infusion and for at least 2 hours thereafter, and laboratory tests were obtained at baseline and every 2 weeks thereafter. No infusion reactions were documented throughout treatment—specifically, there was no fever, chills, rash, dyspnea, hypotension, or other acute infusion-related events. Hematologic and biochemical parameters were clinically unremarkable; at most, a transient, mild elevation in total bilirubin was observed, peaking at 20 μmol/L, which resolved spontaneously without intervention, corticosteroid administration, hospitalization, or interruption of therapy.

## Discussion

EGFR-TKI resistance represents a central challenge in clinical practice. Studies have demonstrated that nearly all patients receiving targeted therapy will inevitably experience disease progression due to tumor heterogeneity-driven clones arising from resistance, which may precisely account for the observed suboptimal real-world outcomes (4, 9). EGFR-TKI resistance can be categorized into intrinsic resistance (progression within 3 months of initial treatment) and acquired resistance (progression following an initial response) (5). Approximately 70%-80% of clinically resistant patients fall into the acquired resistance category, rendering it a key focus of current clinical research. The mechanisms underlying resistance are intricate. The most prevalent resistance mechanism for first/second-generation EGFR-TKIs is the T790M secondary mutation (accounting for over 50%), whereas third-generation EGFR-TKIs like osimertinib exhibit more diverse resistance mechanisms. Notably, the resistance mechanisms remain unclear in 40%-50% of first-line osimertinib-resistant patients and 30%-40% of second-line resistant patients. These “unknown-mechanism resistance” patients often resort to empirical chemotherapy combined with anti-angiogenic therapy, lacking precise treatment strategies (10). Researchers have conducted extensive investigations to explore therapeutic strategies post-resistance. In recent years, clinical studies on osimertinib rechallenge, combination with chemotherapy, and combination with TKIs targeting distinct pathways have been initiated. However, due to unidentified resistance targets and clonal escape driven by tumor spatial heterogeneity, the efficacy of these approaches in patients with unclear resistance mechanisms remains limited (11–13).

Against the above background, this case report describes a patient with advanced EGFR-mutant lung adenocarcinoma who developed acquired resistance to osimertinib with unknown mechanisms. The patient received ACTL therapy and ultimately achieved a cCR and maintained it for 6 years of follow-up. This case confirms that ACTL can break through the barrier of tumor heterogeneity and eliminate drug-resistant clones unrecognizable by traditional techniques. According to previous studies and this case, the acquired resistance to EGFR-TKIs is to some extent associated with the immunosuppressive state of the TME (14). ACTL not only achieves the remodeling and reversal of the immune microenvironment during treatment, but the long-term immune effect it induces also provides new evidence for the “curative treatment” of advanced lung cancer.

**Figure 4:**
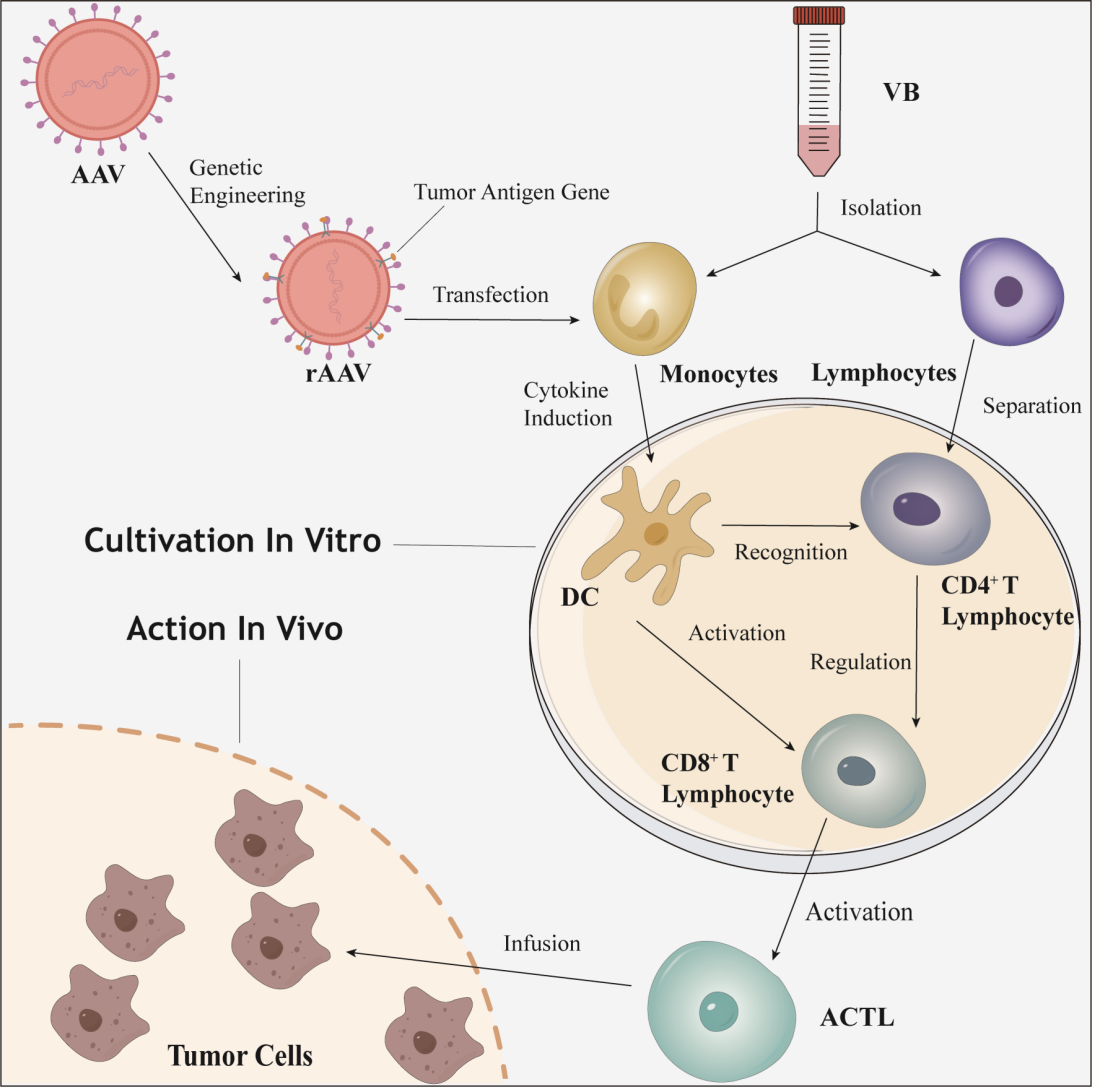
The preparation and mechanism of action diagram of ACTL. ACTL, antigen-specific cytotoxic T lymphocyte; AAV, adeno-associated virus; rAAV, recombinant adeno-associated virus; VB, venous blood; DC, dendritic cell.

Tumor immunotherapy is the “fourth pillar” of cancer treatment. ACT, a key component of immunotherapy, achieves precise targeting of tumor cells by processing and reinfusing patients’ autologous immune cells ex vivo. T cell lines are the cornerstone of the conventional immune system and important tools for ACT. According to cell modification strategies, ACT can be divided into non-genetically modified therapies [such as tumor-infiltrating lymphocyte (TIL), cytokine-induced killer cell (CIK)] and genetically modified therapies (such as CAR-T, TCR-T). The former has high safety but insufficient targeting, while the latter has high precision but carries risks of cytokine release syndrome (CRS) and “on-target, off-tumor” toxicity (15). ACTL therapy represents an innovative breakthrough in the field of ACT. The specific preparation process is shown in **Figure 4**. This process does not require gene editing; instead, it achieves precise recognition of tumor antigens through the natural interaction between T cell receptors (TCRs) and MHC molecules. It not only avoids the risks of genetic modification and toxicity associated with CAR-T/TCR-T therapies but also addresses the insufficient targeting of TILs and CIK cells, thus providing a balanced option for solid tumor treatment that lies between fully natural T cells and genetically engineered T cells (7).

The downstream cells of ACTL are CTLs similar to those in the conventional human immune system. However, ACTL enhances immune activation *in vitro* and promotes the clonal expansion of CTLs by forcing the efficient expression and presentation of target antigens through tumor antigen genes. This ensures that the number of reinfused ACTL cells far exceeds that of spontaneously activated effector T cells *in vivo*, while avoiding the inhibition of T cell activation by TME (such as the premature exhaustion of the PD-1/PD-L1 pathway) (16, 17). In addition to the direct antigen-specific killing effect, ACTL can also reverse the immunosuppressive state of the TME through microenvironment remodeling, establishing long-term immune surveillance to prevent clonal escape caused by tumor heterogeneity (7, 18–21). Compared with traditional ACT technologies, the innovation of ACTL lies in its dual strategy of “natural antigen presentation + ex vivo directional activation,” which balances safety (no CRS or off-target toxicity), targeting (antigen-specific recognition), and durability of efficacy (formation of immune memory). This “gene-editing-free” precise immune activation model not only provides a new treatment option for refractory lung cancers such as EGFR-TKI-resistant cases but also expands the application boundaries of solid tumor immunotherapy.

It is worth noting that two other reported indicators in this patient, IFN-γ and the CD4^+^/CD8^+^ ratio, as important markers reflecting immune status, their dynamic changes revealed a close association between the immune microenvironment and treatment response. At initial diagnosis, the patient’s IFN-γ and CD4^+^/CD8^+^ ratio were within normal ranges, suggesting that although the immune system was not completely dysfunctional, the tumor had formed metastatic lesions through immune escape mechanisms. With the emergence of EGFR-TKI treatment resistance, the IFN-γ level dropped from 11.2 pg/ml to 6.1 pg/ml, and the CD4^+^/CD8^+^ ratio plummeted from 1.4 to 0.9. This change indicated the exacerbation of immunosuppressive status in the TME—IFN-γ, as a key immunoregulatory factor, its decline may weaken T cell-mediated antitumor activity and antigen presentation efficiency; the imbalance of the CD4^+^/CD8^+^ ratio suggested the dysfunction of helper T cells and the exhaustion of cytotoxic T cells (22–24). After ACTL treatment, the patient’s immune status dramatically reversed: the IFN-γ level surged to 32.4 pg/ml, and the CD4^+^/CD8^+^ ratio rose to 6.2, accompanied by significant regression of tumor lesions and eventual achievement of cCR. The supraphysiological elevation of the CD4^+^/CD8^+^ ratio after treatment may indicate enhanced immune memory effects induced by ACTL, laying the foundation for the patient’s long-term cCR (25).

This phenomenon indirectly reflects the potential of ACTL to reshape the immune microenvironment, transforming a “cold tumor” into a “hot tumor”. Currently, there is a lack of research or validation on the precise downstream mechanisms of ACTL, but we can infer from the two indicators described in this case—the surge in IFN-γ and the reshaping of the CD4^+^/CD8^+^ ratio—that the mechanism by which ACTL therapy reshapes the TME involves reversing the immunosuppressive microenvironment to an immune-activated state. The surge in IFN-γ levels can not only enhance tumor antigen presentation by upregulating MHC class I molecule expression and inhibit angiogenesis to limit tumor nutrient supply, but also induce tumor cell autophagy. Meanwhile, IFN-γ polarizes the phenotype of immune cells, promoting the differentiation of M1 macrophages and mature DC cells, inhibiting immunosuppressive cells such as Tregs and MDSCs, and chemotaxing effector T cell infiltration to form an “immunoinflammatory” microenvironment (26–28). The jump in the CD4^+^/CD8^+^ ratio reflects the dominance of a Th1-type immune response, in which CD4^+^ T cells assist in the activation and proliferation of CD8^+^ T cells and the formation of memory T cells, enhancing antibody-mediated ADCC effects (29, 30). Additionally, the two indicators have a bidirectional regulatory synergistic effect: IFN-γ enhances Th1 polarization of CD4^+^ T cells, and CD4^+^ T cells secrete IL-2 to further promote IFN-γ release, jointly counteracting immunosuppressive factors in the TME such as lactic acid accumulation and high PD-L1 expression, and reshaping blood vessel structures to improve immune cell infiltration efficiency (31, 32). It is possible that the changes in these two indicators are merely the results or phenomena of deeper internal mechanisms, but their changes are important evidence of immune microenvironment changes, which must also have a considerable impact on downstream factors or the microenvironment.

ACTL demonstrated a favorable safety profile throughout treatment: no infusion reactions occurred, and the transient post-infusion fluctuations in blood parameters returned to the normal range. This contrasts with the toxicity spectra and pronounced post-treatment reactions reported with other ACT (15). The safety may relate to several features of ACTL: first, ex vivo activation/expansion without lymphodepletion; second, no need for systemic IL-2 support after infusion; and third, non-genetic, antigen-specific priming via rAAV-transfected dendritic cells. Together, these factors may reduce the risk of CRS or severe organ toxicities associated with cellular heterogeneity. For future prospective studies, pre-specifying hold/resume thresholds (e.g., for alanine/aspartate aminotransferase or bilirubin) and standardized management algorithms will help harmonize reporting and strengthen comparisons across centers. These findings support the view that, in patients with EGFR-TKI-resistant non-small cell lung cancer, ACTL can deliver meaningful clinical benefit with manageable toxicity and warrants broader evaluation in multicenter settings.

In the exploration of EGFR-TKI resistance treatment, multiple clinical studies have provided comparisons and supplements for the value of ACTL therapy. The FLAURA2 study confirmed that although osimertinib combined with chemotherapy prolonged mPFS from 12.5 months to 23.5 months, the addition of chemotherapy was accompanied by superimposed toxicities such as bone marrow suppression and gastrointestinal reactions, and did not address the problem of drug-resistant clone evolution caused by tumor heterogeneity (33). Subgroup analysis of the Mariposa study in Asian populations showed that the amivantamab-lazertinib bispecific antibody regimen significantly prolonged mPFS compared with osimertinib monotherapy (14.4 months *vs*. 11.1 months), but its efficacy was limited to drug-resistant subtypes with EGFR/c-MET dual-pathway co-dependency (34). The INSIGHT 2 study focused on populations with MET abnormalities and found that MET-TKI combined with osimertinib achieved an ORR of 49%–52% in patients with high MET amplification, but had limited efficacy in patients with “unknown mechanism resistance” without clear targets (ORR<30%) (35). Due to the unclear resistance mechanism in this case, the patient could hardly benefit from traditional targeted regimens, while ACTL activated CTLs by targeting broad-spectrum tumor antigens, bypassing dependence on specific genetic variations, achieving deep remission in this patient and filling the existing treatment gap. Additionally, Wang et al. reported that after ACTL treatment in 3 NSCLC patients with brain metastases, the brain metastases completely resolved and the overall survival exceeded 2 years (36); Hong et al.’s study confirmed that ACTL can completely resolve untreated melanoma brain metastases, highlighting ACTL’s unique advantage of precise killing through antigen-specific CTLs (37). ACTL provides a “driver gene mutation-independent” solution for advanced NSCLC patients with unknown mechanism resistance and multi-line treatment failure.

Although this case demonstrates the remarkable efficacy of ACTL therapy in patients with EGFR-TKI-resistant progression, as a single-center, small-sample study, it still has the following limitations in medical practice and mechanistic exploration. Efficacy in this case was only evaluated by imaging and serum tumor markers (RECIST 1.1 criteria), lacking dynamic biopsy data of the tumor microenvironment (such as immune cell infiltration profiles, PD-L1 expression) before and after treatment, making it difficult to accurately analyze the immune mechanisms by which ACTL induces clinical complete response (such as memory T cell subtype distribution, cytokine network changes). The determination of “unknown mechanism resistance” was only based on known gene mutation testing, which may have missed rare resistance pathways (such as abnormal epigenetic regulation or non-coding RNA-driven mechanisms). Additionally, the ACTL treatment cycle (once every two weeks) and duration of treatment (maintenance therapy after cCR) lack supporting data from prospective studies and are primarily determined by clinical experience, requiring further optimization. Therefore, this therapy requires further validation of its efficacy through multi-center, prospective clinical studies, while simultaneously optimizing antigen screening strategies using technologies such as single-cell sequencing and organoid models, to promote its transformation from case-based experience to standardized treatment.

This case is the first to confirm that ACTL cellular immunotherapy has significant efficacy in patients with advanced lung adenocarcinoma who have EGFR-TKI resistance with unknown mechanisms, and can realize the functional reversal of the immune microenvironment from a “cold tumor” to a “hot tumor” through reshaping it. Although this study still requires validation with larger sample sizes and longer follow-up data, it provides the first evidence of “clinical cure by immunotherapy monotherapy” for advanced lung cancer with EGFR-TKI acquired resistance, offering a new paradigm for overcoming the clinical challenge of targeted therapy resistance. With the iterative upgrading of immunotherapy technologies, the integrated strategy of “targeting-immunity-memory” is expected to redefine the treatment landscape of advanced lung cancer and promote the field from “survival extension” to “functional cure”.

## Data Availability

The original contributions presented in the study are included in the article/supplementary material. Further inquiries can be directed to the corresponding authors.
